# Epstein–Barr Virus Infection Is Associated with an Elevated Tumor–Stroma Ratio and Older Age in Oral Squamous Cell Carcinoma

**DOI:** 10.3390/v18020241

**Published:** 2026-02-14

**Authors:** Eris Nurul Rahmadhini, Irna Sufiawati, Hasrayati Agustina, Okky Husain, Seto Adiantoro Sadputranto, Adi Idris

**Affiliations:** 1 Oral Medicine Residency Program, Faculty of Dentistry, Universitas Padjadjaran, Bandung 40132, Indonesia; erisnurulrahmadhini@gmail.com; 2 Department of Oral Medicine, Faculty of Dentistry, Universitas Padjadjaran, Bandung 40132, Indonesia; 3 Dr. Hasan Sadikin General Hospital, Bandung 40161, Indonesia; hasrayati@unpad.ac.id (H.A.); okky.husain@gmail.com (O.H.); seto.spbm@gmail.com (S.A.S.); 4 Department of Anatomical Pathology, Faculty of Medicine, Universitas Padjadjaran, Bandung 40161, Indonesia; 5 Department of Oral Maxillofacial Surgery, Faculty of Dentistry, Universitas Padjadjaran, Bandung 40132, Indonesia; 6 Center for Immunology and Infection Control, School of Biomedical Sciences, Queensland University of Technology, Brisbane, QLD 4001, Australia; a2.idris@qut.edu.au

**Keywords:** Epstein–Barr virus, oral squamous cell carcinoma, tumor microenvironment, tumor stroma ratio, tumor-infiltrating lymphocytes, clinicopathological features

## Abstract

Epstein–Barr virus (EBV) is an oncogenic virus implicated in several epithelial malignancies; however, its role in the tumor microenvironment of oral squamous cell carcinoma (OSCC) remains unclear. This study investigated the association between EBV infection and clinicopathological and microenvironmental features of OSCC. A cross-sectional analysis was conducted on 62 archived OSCC biopsy specimens. EBV was detected using polymerase chain reaction (PCR), and clinical data were obtained from medical records. Tumor–stroma ratio (TSR), perineural invasion (PNI), lymphovascular invasion (LVI), and histological differentiation were assessed microscopically, while tumor-infiltrating lymphocytes (TILs) were quantified using ImageJ software version 1.54j (National Institutes of Health, Bethesda, MD, USA). EBV DNA was identified in 43.5% of cases. EBV positivity was significantly associated with older age (*p* = 0.046), especially among patients aged 60 years or older. All EBV-positive tumors exhibited a high tumor–stroma ratio, which was significantly associated with EBV status (*p* = 0.031). No significant associations were observed between EBV status and sex, tumor site, clinical stage, TILs, PNI, LVI, or histological differentiation. These findings indicate that EBV-positive OSCC is characterized by distinct microenvironmental features, particularly an elevated tumor–stroma ratio, and suggest a potential role for EBV status in microenvironmental profiling and prognostic stratification.

## 1. Introduction

Oral squamous cell carcinoma (OSCC) accounts for approximately 90% of oral cancers and represents the most prevalent oral malignancy [1,2,3,4]. Major risk factors include tobacco use, alcohol consumption, betel nut chewing, ultraviolet radiation, genetic and nutritional factors, and viral infections [1].

Epstein–Barr virus (EBV) is a ubiquitous oncogenic herpesvirus with a well-established role in several epithelial and lymphoid malignancies. EBV infection of oral epithelial cells has been demonstrated in experimental models, suggesting that the virus is capable of infecting and persisting within oral squamous cells [5]. In addition, molecular studies have shown that EBV may influence epithelial cell behavior through delayed differentiation, epigenetic reprogramming, and metabolic alterations, including activation of the Warburg effect, which are implicated in cancer-related pathways such as cell proliferation, migration, invasion, and resistance to apoptosis [6].

In clinical settings, EBV DNA has been detected in a subset of oral and oropharyngeal squamous cell carcinomas, with meta-analyses reporting a relatively high prevalence of EBV infection in these tumors compared with non-cancer controls [7]. Co-infection with EBV and HPV has been detected in 15–20% of carcinomas occurring in the oropharynx [8,9,10]. In a previous systematic review and meta-analysis, EBV and HPV co-infection was identified in a subset of oral and oropharyngeal squamous cell carcinomas, which suggests a biological interaction between oncogenic viruses in head and neck cancers [11]. Nevertheless, the clinical and histopathological relevance of EBV infection in OSCC, independent of or in conjunction with other viral infections, has not been fully elucidated.

The prognosis for oral cancer patients remains unfavorable, with the 5-year survival rate for oral cancer patients being below 50%, dropping to less than 30% in advanced stages, and not significantly improving in recent decades. Many factors, including EBV infection in oral cancer, are associated with increased tumor aggressiveness, which negatively impacts prognosis by facilitating tumor development [7,8]. Important factors related to the survival of cancer patients can include clinical, such as age, gender, cancer location, cancer stage, and histopathological parameters, including tumor-infiltrating lymphocytes (TILs), tumor–stroma ratio (TSR), perineural invasion (PNI), lymphovascular invasion (LVI), and degree of differentiation. Clinical and histopathological parameters are very useful in accurately assessing the biological aggressiveness of oral cancer and enabling the development of customized treatment plans for each specific cancer [12,13,14].

Understanding how EBV infection influences the tumor microenvironment provides valuable insights into tumor immunobiology and may uncover new therapeutic vulnerabilities. EBV-driven modulation of immune infiltration, stromal composition, and metabolic pathways could affect disease progression and treatment response. Therefore, this study aimed to analyze the association between EBV status and clinicopathological as well as microenvironmental parameters in oral squamous cell carcinoma, highlighting its potential relevance as a biomarker and therapeutic target.

## 2. Materials and Methods

### 2.1. Study Design and Sample Selection

This study utilized a comparative analytical design with a cross-sectional approach. Patients aged 18 years or older with oral cavity cancer who had not received prior chemotherapy or radiotherapy were screened for eligibility according to predefined inclusion criteria. Fresh tumor tissue specimens were initially used for EBV detection by polymerase chain reaction (PCR). The same specimens were then processed into formalin-fixed, paraffin-embedded (FFPE) blocks and subjected to hematoxylin and eosin (H&E) staining to confirm the diagnosis of OSCC and to assess histopathological parameters, including TILs, TSR, PNI, LVI, and degree of differentiation. Cases with damaged, insufficient, or unreadable tissue specimens were excluded.

Based on these criteria, a total of 62 OSCC cases met the inclusion requirements and were included in the final analysis. All samples were anonymized before analysis, and the study was conducted in accordance with institutional ethical standards.

### 2.2. EBV Detection

Genomic DNA was extracted from fresh tumor tissue using standard laboratory protocols. Epstein–Barr virus (EBV) DNA detection was performed with a commercial multiplex real-time PCR assay (Novaplex™ EBV assay, Seegene, Seoul, Republic of Korea) in accordance with the manufacturer’s instructions. PCR amplification and detection were carried out on a Bio-Rad Opus CFX96 real-time PCR system (Bio-Rad Laboratories, Hercules, CA, USA).

The assay utilizes proprietary primer and probe sets that target EBV-specific genomic regions and other Herpesviridae family members. Each PCR run included internal, positive, and negative controls supplied by the manufacturer. EBV positivity was determined based on amplification curves and cycle threshold (Ct) values following the predefined criteria outlined in the assay manual. Human papillomavirus (HPV) status was assessed separately using a dedicated HPV PCR assay as part of routine molecular diagnostics.

In this study, EBV positivity was defined as the presence of EBV DNA, either as a single infection or in combination with HPV. Cases exhibiting HPV infection without EBV were excluded from the analysis, while the presence of other non-HPV viruses detected by the multiplex assay was not included in the analytical model. To ensure transparency, the distribution of EBV-only cases and EBV–HPV co-infected cases is reported separately in the Section 3.

### 2.3. Clinical Parameters

Clinical parameters, including age, gender, tumor location, and cancer stage, were extracted from patients’ medical records and radiological evaluations employing computed tomography (CT) scans. Age was categorized into four groups for descriptive and analytical purposes: 18–30 years, 31–45 years, 46–60 years, and >60 years, based on clinical relevance.

### 2.4. Histopathological Assessment

Histopathological analyses were performed using stored biological material (SBM) in the form of formalin-fixed, paraffin-embedded (FFPE) tumor tissue sections. Hematoxylin and eosin (H&E)-stained slides were reviewed by an experienced anatomical pathologist who was blinded to EBV status.

**Figure 1:**
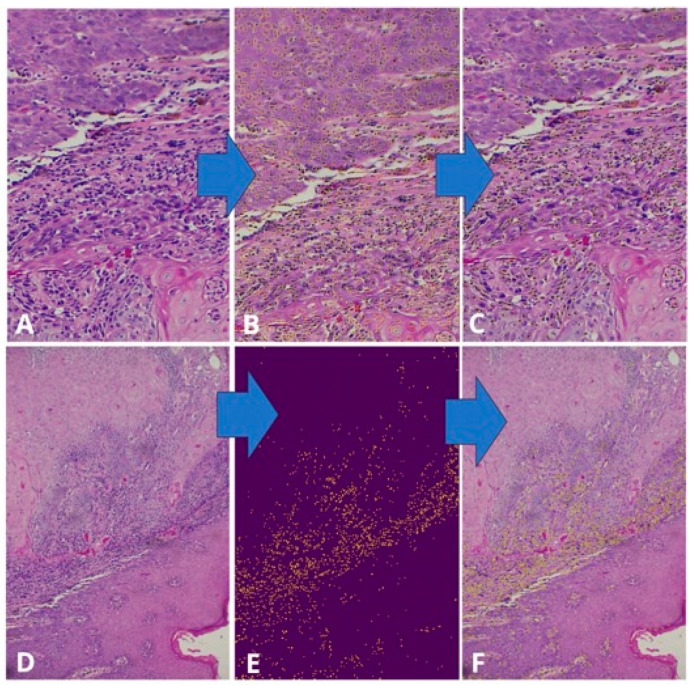
Quantitative assessment of TILs using ImageJ. Nuclei detection was performed by identifying maximum points, followed by clustering and selection at higher magnification. Blue arrows indicate the sequential steps of the image-processing workflow from the original micrograph to particle detection. (**A**–**C**) Yellow dotted contours mark the selected region of interest. (**D**,**E**) Comparison between the original image and the distribution of detected particles. (**F**) The particle outline is superimposed on the original image, showing a yellow line encircling the lymphocytes.

Tumor–stroma ratio (TSR) was evaluated at the invasive tumor front. TSR was classified as high when tumor cells occupied more than 50% of the selected microscopic field (stroma-poor tumors) and low when stromal components occupied more than 50% of the field (stroma-rich tumors). Perineural invasion (PNI) and lymphovascular invasion (LVI) were recorded as present or absent based on established histopathological criteria. Degree of differentiation was classified as well-, moderately-, or poorly differentiated according to Broders’ criteria. Quantitative assessment of tumor-infiltrating lymphocytes (TILs) was performed on H&E-stained sections using ImageJ software (National Institutes of Health, Bethesda, MD, USA). TILs were quantified as the proportion of stromal area occupied by mononuclear inflammatory cells following standardized evaluation guidelines (Figure 1).

### 2.5. Statistical Analysis

Statistical analyses were conducted using SPSS Version 30.0 for Windows (IBM Corp., Armonk, NY, USA). Categorical variables, such as age, sex, tumor location, TSR, PNI, LVI, and cancer stage, were analyzed using either the Chi-square test or Fisher’s exact test, as appropriate. Results for these variables are reported as frequencies and percentages. The Shapiro–Wilk test assessed the normality of the TIL data. Because the data were not normally distributed, the Mann–Whitney U test was used for comparisons, with results reported as median (minimum–maximum) values. All statistical tests were two-tailed, and statistical significance was defined as *p* < 0.05.

## 3. Results

A total of 62 oral cancer samples were analyzed, of which 27 (43.5%) were EBV-positive, and 35 (56.5%) were EBV-negative. Among the EBV-positive cases, 17 samples (63.0%) showed EBV infection alone, while 10 samples (37.0%) demonstrated EBV co-infection with other viruses, including cytomegalovirus (CMV), human herpesvirus-7 (HHV-7), and high-risk human papillomavirus (HPV) types 16 and 33. Samples positive for HPV alone without EBV were excluded from the analysis.

### 3.1. Clinical Characteristics

**Figure 2:**
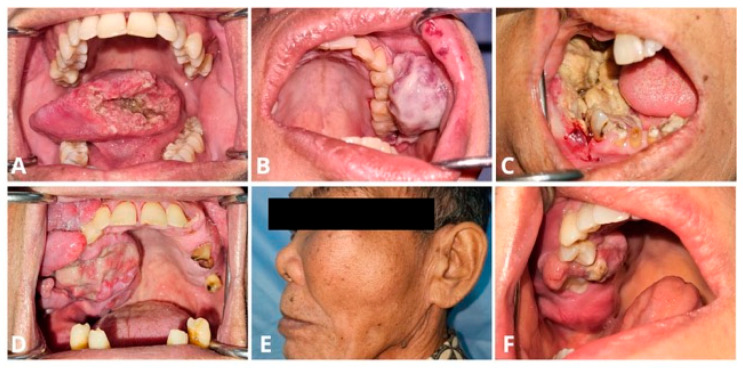
Clinical presentation of tumors according to the anatomical site. (**A**) Tumor of the tongue; (**B**) Tumor of the buccal mucosa; (**C**) Tumor of the mandible; (**D**) Tumor of the palate; (**E**) Tumor of the zygoma; (**F**) Tumor of the maxilla.

The distribution of clinical characteristics according to EBV status is summarized in Table 1. A significant association was observed between EBV status and age (*p* = 0.046). EBV-positive cases were more frequently identified in patients aged over 60 years (37.0%) compared with EBV-negative cases (11.4%). No significant associations were found between EBV status and gender (*p* = 0.585), tumor site (*p* = 0.239), or cancer stage (*p* = 0.406). Although not statistically significant, male patients constituted a slightly higher proportion of EBV-positive cases (55.6%) compared with EBV-negative cases (48.6%). The tongue was the most common tumor site overall, accounting for 41.9% of cases, and 44.4% of EBV-positive tumors (Figure 2). Advanced disease was predominant in both groups, with stage 4a being the most frequent stage among EBV-positive cases (64.0%).

### 3.2. Histopathological Parameters

**Figure 3:**
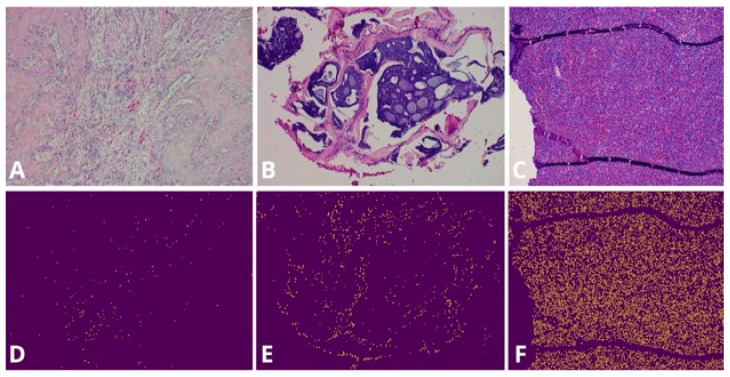
Quantification of TILs. (**A**–**C**) Original hematoxylin and eosin (H&E)-stained micrographs. (**D**–**F**) Corresponding images showing the distribution of lymphocyte particles.

Histopathological findings according to EBV status are presented in Table 2. The median TIL count was slightly higher in EBV-positive cases (median 467; range 78–1221) than in EBV-negative cases (median 392; range 73–1463); however, this difference was not statistically significant (*p* = 0.994) (Figure 3).

**Figure 4:**
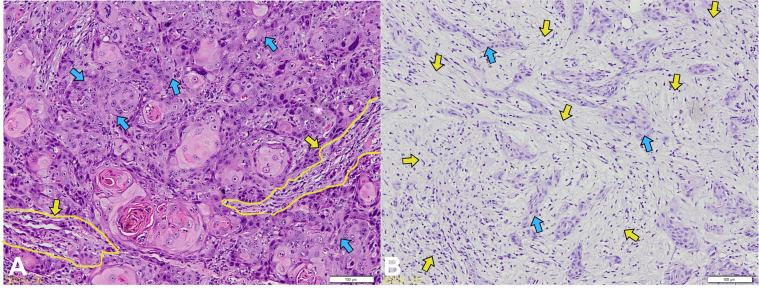
Representative H&E-stained micrographs showing the TSR. Tumor–stroma ratio image. (**A**) High TSR (tumor > 50%); (**B**) Low TSR (tumor ≤ 50%); Yellow arrows and yellow circles highlight the stromal regions within the selected field; blue arrows indicate tumor regions.

In contrast, a significant association was observed between EBV status and TSR (*p* = 0.031). All EBV-positive tumors exhibited a high TSR (100%), whereas 82.9% of EBV-negative tumors showed a high TSR (Figure 4).

**Figure 5:**
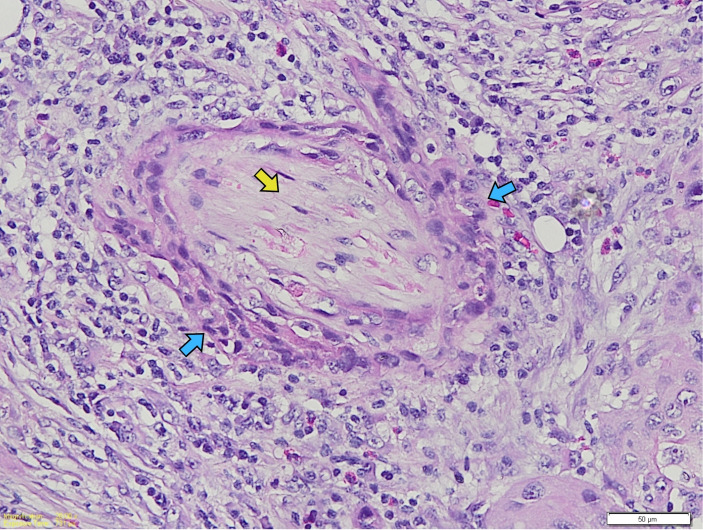
Representative micrograph showing PNI. The blue arrow indicates the tumor, while the yellow arrow indicates the involved nerve.

**Figure 6:**
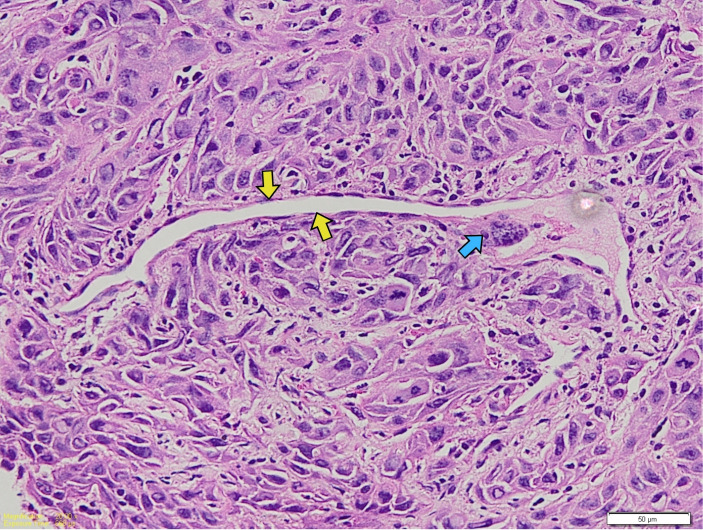
Representative micrograph showing LVI. The tumor compresses vascular endothelial cells (yellow arrows) and invades the vascular lumen (blue arrows).

No significant associations were identified between EBV status and PNI (*p* = 0.308) or lymphovascular invasion (LVI) (*p* = 0.136). Nevertheless, the proportions of PNI (22.2% vs. 11.4%) and LVI (37.0% vs. 20.0%) were higher in EBV-positive cases compared with EBV-negative cases (Figure 5 and Figure 6).

**Figure 7:**
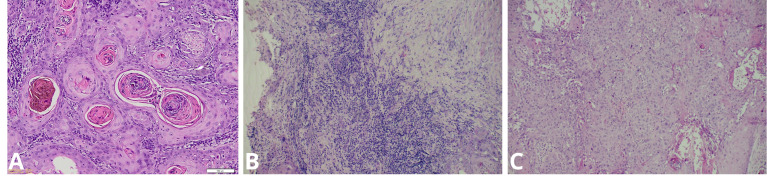
Representative H&E-stained micrographs illustrating the degree of differentiation in EBV-positive cases. (**A**) Well-differentiated tumor with keratin pearls; (**B**) Moderately differentiated; (**C**) Poorly differentiated.

Tumor differentiation grade did not differ significantly according to EBV status (*p* = 0.584). Moderately differentiated tumors were more common among EBV-positive cases (40.0%) compared with EBV-negative cases (25.0%), whereas well-differentiated tumors predominated in the EBV-negative group (60.0%) (Figure 7).

## 4. Discussion

This study showed that EBV DNA was found in 43.5% of oral squamous cell carcinoma (OSCC) cases, which means that EBV infection is relatively common in this group. This prevalence aligns with previous meta-analyses indicating EBV detection rates of roughly 40–50% in oral and oropharyngeal squamous cell carcinomas [7]. However, the present findings should be interpreted as an association rather than evidence of a causal role of EBV in oral carcinogenesis.

Among the clinical parameters evaluated, age was the only factor significantly associated with EBV status. EBV-positive tumors were more frequently observed in patients older than 60 years. This finding may be attributable to age-related immunosenescence, chronic inflammation, and cumulative genomic alterations, all of which can facilitate viral persistence or reactivation [15,16,17,18]. Previous studies have demonstrated that aging is accompanied by impaired immune surveillance and epigenetic changes, such as DNA hypermethylation, which may influence host–virus interactions [18,19]. However, the cross-sectional design of this study limits the ability to conclude temporal or causal relationships.

No significant associations were identified between EBV status and gender, tumor location, or cancer stage. Although a slightly higher proportion of males was observed among the EBV-positive cases, this difference did not reach statistical significance and may have been influenced by behavioral or environmental factors rather than virus-specific effects [20,21]. The tongue was the most frequent tumor site in both the EBV-positive and EBV-negative groups, consistent with prior reports. Keratinized squamous epithelium on the tongue, especially the lateral edges and base, has numerous CD21 receptors and lymphoid tissue, facilitating EBV entry [22]. Advanced-stage disease predominated in this cohort, which may reflect delayed diagnosis and healthcare access rather than differences related to viral status [23].

Regarding histopathological parameters, median TIL levels were numerically higher in the EBV-positive tumors, although this difference did not reach statistical significance. Increased lymphocytic infiltration may reflect host immune recognition of viral antigens; however, effective antitumor immunity may be compromised by EBV-associated immune evasion mechanisms, including the modulation of immune checkpoint pathways, such as the PD-1/PD-L1 pathway, as reported in previous studies [19]. These findings underscore the complex interplay between viral infection and immune response within the tumor microenvironment.

A main finding of this study is the strong association between EBV status and TSR, as all EBV-positive tumors exhibited a high TSR. The TSR quantifies the proportion of tumor cells relative to stromal components within the tumor microenvironment and is recognized as a reliable histopathological marker associated with tumor behavior in head and neck squamous cell carcinoma, as supported by recent systematic reviews and meta-analyses [24]. Although evidence regarding the relationship between EBV infection and TSR in OSCC remains limited, prior studies suggest that EBV may modulate tumor metabolism and the composition of the microenvironment, including increased glycolytic activity consistent with the Warburg effect and altered tumor–stroma interactions [6,25]. The Warburg effect has been associated with tumor–microenvironment interactions in experimental cancer models [6]. These mechanisms may contribute to a stroma-poor tumor phenotype and affect tumor growth dynamics as well as the distribution of immune cells within the tumor microenvironment [6,25]. However, these biological processes were not directly assessed in the present study and should be interpreted with caution. Collectively, the results indicate that the observed association between EBV status and a high TSR is biologically plausible based on existing experimental evidence, although interpretative limitations of this study remain.

No statistically significant associations were observed between EBV status and perineural invasion (PNI) or lymphovascular invasion (LVI). Although higher proportions of PNI and LVI were observed in EBV-positive tumors, these differences did not reach statistical significance and may be influenced by sample size limitations. Previous studies have reported that EBV-related signaling pathways, including NF-κB and JAK/STAT activation, may promote invasive tumor behavior [6,18,26]. However, the present findings do not support a definitive association between EBV status and these invasive features in OSCC.

Regarding tumor differentiation, no significant relationship was observed between EBV status and degree of differentiation. While a higher proportion of EBV-positive tumors exhibited well or moderately differentiated morphology, this observation did not reach statistical significance and is consistent with prior reports indicating variable associations between EBV infection and tumor differentiation in OSCC [7,27]. Immunological and microenvironmental factors may contribute to these patterns, although further investigation is required [28].

This study has several limitations. EBV detection relies on PCR analysis of tumor tissue, which does not allow for the localization of EBV infection within specific cell populations. As a result, it is unclear whether EBV DNA originated from malignant epithelial cells, infiltrating EBV-infected lymphocytes, or viral particles. The lack of EBER in situ hybridization is a significant limitation. Additionally, although HPV status was assessed, the small sample size prevented stratified analyses to fully evaluate the independent or combined effects of viral co-infection on TSR.

Despite these limitations, this study highlights an association between EBV infection and specific clinicopathological features, particularly older age and a high tumor-stroma ratio, in OSCC. These results deepen the understanding of potential virus-associated tumor microenvironmental characteristics and support the need for future studies incorporating cell-specific viral localization, molecular profiling, and larger patient cohorts to further clarify the role of EBV in oral squamous cell carcinoma.

## 5. Conclusions

This study indicates that EBV DNA was detected in a substantial proportion of OSCC cases. EBV positivity was significantly associated with older patient age and a high tumor–stroma ratio, suggesting that EBV infection may be linked to distinct clinicopathological and tumor microenvironment characteristics in OSCC. Other clinical and histopathological parameters, including tumor-infiltrating lymphocytes, perineural invasion, lymphovascular invasion, degree of differentiation, and cancer stage, were not significantly associated with EBV status.

Although these findings do not establish a causal role for EBV in oral carcinogenesis, they highlight a potential association between EBV presence and specific microenvironmental features. Given the limitations of PCR-based EBV detection and the absence of cell-specific viral localization, further studies incorporating EBER in situ hybridization, molecular profiling, and larger cohorts are warranted. Improved understanding of the relationship between EBV infection and the tumor microenvironment may help refine future risk stratification and the biological characterization of OSCC.

## Figures and Tables

**Table 1:** The association between EBV status and clinical parameters.

Clinical Parameters	Total *n* = 62	EBV Status		*p* Value
		Positive *n* = 27	Negative *n* = 35	
	*n* (%)	*n* (%)	*n* (%)	
Gender				0.585
Male	32 (51.6)	15 (55.6)	17 (48.6)	
Female	30 (48.4)	12 (44.4)	18 (51.4)	
Age (years)				0.046 *
18–30	5 (8.1)	2 (7.4)	3 (8.6)	
31–45	12 (19.4)	2 (7.4)	10 (28.6)	
46–60	31 (50.0)	13 (48.1)	18 (51.4)	
>60	14 (22.6)	10 (37.0)	4 (11.4)	
Site				0.239
Mandible	14 (22.6)	3 (11.1)	11 (31.4)	
Palate	7 (11.3)	4 (14.8)	3 (8.6)	
Buccal Mucosa	5 (8.1)	3 (11.1)	2 (5.7)	
Tongue	26 (41.9)	12 (44.4)	14 (40)	
Oropharynx	4 (6.5)	3 (11.1)	1 (2.9)	
Maxilla	3 (4.8)	0 (0)	3 (8.6)	
Gingiva	2 (3.2)	1 (3.7)	1 (2.9)	
Zygoma	1 (1.6)	1 (3.7)	0 (0)	
Cancer Staging				0.406
Stage 1	2 (3.6)	0 (0)	2 (6.5)	
Stage 2	6 (10.7)	4 (16)	2 (6.5)	
Stage 3	7 (12.5)	2 (8)	5 (16.1)	
Stage 4a	36 (64.2)	16 (64)	20 (64.5)	
Stage 4c	5 (8.9)	3 (12)	2 (6.5)	

Notes: * *p* < 0.05 was considered statistically significant.

**Table 2:** Association between EBV status and histopathological parameters.

Histopathological Parameters	EBV-Status		*p* Value
	Positive *n* = 27	Negative *n* = 35	
	*n* (%)	*n* (%)	
TILs			0.994
Median	467	392	
Min–Max	78–1221	73–1463	
TSR			0.031 *
Low	0 (0)	6 (17.1)	
High	27 (100)	29 (82.9)	
PNI			0.308
Present	6 (22.2)	4 (11.4)	
Absent	21 (77.8)	31 (88.6)	
LVI			0.136
Present	10 (37)	7 (20)	
Absent	17 (63)	28 (80)	
Cancer differentiation grade			0.584
Well differentiated	10 (50)	12 (60)	
Moderately differentiated	8 (40)	5 (25)	
Poorly differentiated	2 (10)	3 (15)	

Notes: * *p* < 0.05 was considered statistically significant.

## Data Availability

The data presented in this study are available on request from the corresponding author due to privacy/ethical restrictions.

## Abbreviations

The following abbreviations are used in this manuscript:

EBV	Epstein–Barr virus
TILs	Tumor-infiltrating lymphocytes
TSR	Tumor-stroma ratio
PNI	Perineural invasion
OSCC	Oral squamous cell carcinoma
PCR	Polymerase chain reaction
SBM	Stored biological material
CT scan	Computed tomography scan
H&E	Hematoxylin and Eosin
SPSS	Statistical product of service solutions
JAK/STAT	Janus Kinase Signal Transducer and Activator of Transcription
